# Continuation Versus De-escalation of Broad-Spectrum Antibiotic Therapy in Critically Ill COVID-19 Patients

**DOI:** 10.1007/s44229-023-00027-0

**Published:** 2023-02-28

**Authors:** Namareq F. Aldardeer, Abeer Nizar A. L. Shukairi, Mohannad E. Nasser, Mohammad Al Musawa, Bayader S. Kalkatawi, Reem M. Alsahli, Aiman M. Elsaed Ramdan, Ismael Qushmaq, Mohammed Aldhaeefi

**Affiliations:** 1 King Faisal Specialist Hospital and Research Centre, Jeddah, Saudi Arabia; 2 Department of Medicine, King Faisal Specialist Hospital and Research Centre, Jeddah, Saudi Arabia; 3 College of Medicine, Alfaisal University, Riyadh, Saudi Arabia; 4 Medication Safety/Clinical Support Pharmacy, King Faisal Specialist Hospital and Research Centre (Gen. Org.), Jeddah, Saudi Arabia; 5 Dr. Erfan & Bagedo General Hospital, Jeddah, Saudi Arabia; 6 Faculty of Medicine, Mansoura University, Mansoura, Egypt; 7 Section of Critical Care Medicine, Department of Medicine, King Faisal Specialist Hospital and Research Center (Gen. Org.), Jeddah, Saudi Arabia; 8 Department of Clinical and Administrative Pharmacy Sciences, College of Pharmacy, Howard University, Washington, DC, USA

**Keywords:** COVID-19, Intensive care unit, De-escalation, Broad-spectrum, Antibiotic

## Abstract

**Background:**

Antibiotic de-escalation (ADE) is a stewardship initiative that aims to reduce exposure to antimicrobials, thus limiting their unwanted effect, including antimicrobial resistance. Our study aims to describe the impact of ADE compared with the continuation of therapy on the outcome of critically ill coronavirus disease 2019 (COVID-19) patients.

**Material and Methods:**

A single-center retrospective study included critically ill COVID-19 adult patients admitted between January 1, 2019 and August 31, 2021, and started on broad-spectrum antibiotics. The primary outcome was intensive care unit (ICU) mortality. In addition, other clinical outcomes were evaluated, including ICU readmissions, length of stay, and superinfection.

**Results:**

The study included 73 patients with a mean age of 61.0 ± 19.4, and ADE was performed in 10 (13.6%) of these. In the ADE group, 8/10 (80%) cultures were positive. ICU mortality was not statistically different between ADE and continuation of therapy groups (60 vs. 41.3%, respectively, *P* = 0.317). Superinfection occurred in 4 (5.4%) patients. Hospital mortality, length of stay, and ICU readmission rates did not differ significantly between groups.

**Conclusion:**

De-escalation of broad-spectrum antibiotics in critically ill covid-19 patients was not associated with higher mortality. A larger cohort is needed to confirm these findings.

**Supplementary Information:**

The online version contains supplementary material available at 10.1007/s44229-023-00027-0.

## Introduction

By the end of January 2022, the pandemic caused by severe acute respiratory syndrome coronavirus (SARS-CoV-2) had exceeded 38 million cumulative confirmed cases and more than 5.5 million cumulative deaths worldwide [1]. The Survival Sepsis Campaign (SSC) published guidelines for the treatment and management of coronavirus disease 2019 (COVID-19) [2]. However, this guideline did not include any recommendations for diagnosing and managing COVID-19 bacterial co-infections [2]. On the other hand, the Infectious Diseases Society of America (IDSA) guidelines highlighted the overuse of antibiotics (AB) in early COVID-19 with a low overall rate of reported co-infection [3]. Data on the rate of secondary bacterial infections in patients with COVID-19 are limited [4]. A meta-analysis aimed to determine the prevalence of confirmed bacterial infections among patients with COVID-19 found that only 3.5% of cases had bacterial co-infection, and 14.3% had secondary bacterial infections [5–9]. Another recent meta-analysis analyzing 31 studies found that the estimated bacterial co-infection was 8.6% (95% CI 4.7–15.2%) [9].

Antibiotic de-escalation (ADE) is a stewardship initiative that aims to reduce exposure to antimicrobials, thus limiting their unwanted effect, including antimicrobial resistance [10]. Many guidelines advocate the daily assessment of de-escalation among septic patients [11, 12]. The international guidelines for sepsis and septic shock suggest daily assessment for the de-escalation of antimicrobials as a weak recommendation based on the very low-quality evidence [2]. A meta-analysis of 12 observational studies and one randomized study showed lower mortality in the ADE arm than control (RR 0.72; 95% CI 0.57–0.91) [9]. However, these results should be interpreted with caution as patients enrolled in the ADE arm were less sick and did not have multidrug-resistant pathogens [9]. A recent study in Greece described lower mortality of patients in the ADE arm than controls despite the high rate of multidrug-resistant pathogens in cultures [13].

During the COVID-19 pandemic, several studies identified an increase in the rate of antibiotic utilization [8, 14, 15]. As a result, many initiatives proposed limiting antibiotics in COVID-19 patients [16, 17]. Notably, the National Institute of Health (NIH) guidelines recommend against antibacterial therapy in moderate to severe COVID-19 unless clinical evidence of infection or secondary infection is suspected [12]. However, data on the ADE of antimicrobials in COVID-19 patients in a critical care setting is limited. Therefore, our study aims to compare the outcome of de-escalation versus the continuation of broad-spectrum antimicrobials in critically ill COVID-19 patients in the setting of a high rate of multidrug-resistant pathogens.

## Materials and Methods

A retrospective study was conducted at King Faisal Specialist Hospital and Research Center-Jeddah (KFSHRC-J), a tertiary care and referral hospital, between January 1, 2019 and August 31, 2021. The hospital institutional review board approved the study (IRB# 2021-65). The study included all COVID-19 patients 18 years or older who required intensive care unit (ICU) admission. Only patients started on the broadest spectrum of antipseudomonal beta-lactams, including carbapenem or ceftazidime-avibactam, were included. Ceftolozane-tazobactam was unavailable at the hospital during the data collection period. The study excluded those who received broad-spectrum antibiotics for less than 48 h, were ICU discharged or died within 48 h of ICU admission. We collected patients' data, including demographics, COVID-19 status, antibiotics used, cultures, baseline labs, and infection sources through the RedCap system. We divided patients according to the broad-spectrum antibiotic course into continuation or ADE therapy groups.

### Study Outcomes

The primary outcome was ICU mortality. Secondary outcomes included superinfection, ICU and hospital length of stay, ICU readmission rate, and hospital mortality.

### Definitions

We defined broad-spectrum antibiotics as antimicrobial therapy covering all relevant pathogens that potentially cause the infectious episode [18]. We considered antipseudomonal carbapenem or ceftazidime-avibactam as our targeted prescribed broad-spectrum antibiotic. We based our broad-spectrum antibiotics choice on the hospital antibiogram, which has a high rate of extended-spectrum antibiotics and multidrug resistance. We defined ADE as replacing a broad-spectrum antibiotic with a narrow spectrum or lower ecological Impact [19, 20]. We considered antibiotics re-escalation as the resumption of a broad-spectrum treatment justified by a clinical worsening, not necessarily related to the initial infection [21]. We defined superinfection as the occurrence of infection with pathogen identification with the need to introduce a new antimicrobial treatment. Finally, we described infection recurrence as the reappearance of an infection after the cessation of all antibiotic therapy [21].

### Statistical Analysis

Data were presented as frequencies and percentages for categorical data and mean ± standard deviation (SD) for continuous data. As appropriate, chi-square or Fisher exact tests were used to test significant differences in categorical variables. We used student *t* tests or Mann–Whitney tests to test significant differences in continuous variables. All *P* values were two-tailed. *P* < 0.05 was considered significant. We used SPSS (Version 25.0. Armonk, NY: IBM Corp) for all statistical analyses.

## Results

**Fig. 1:**
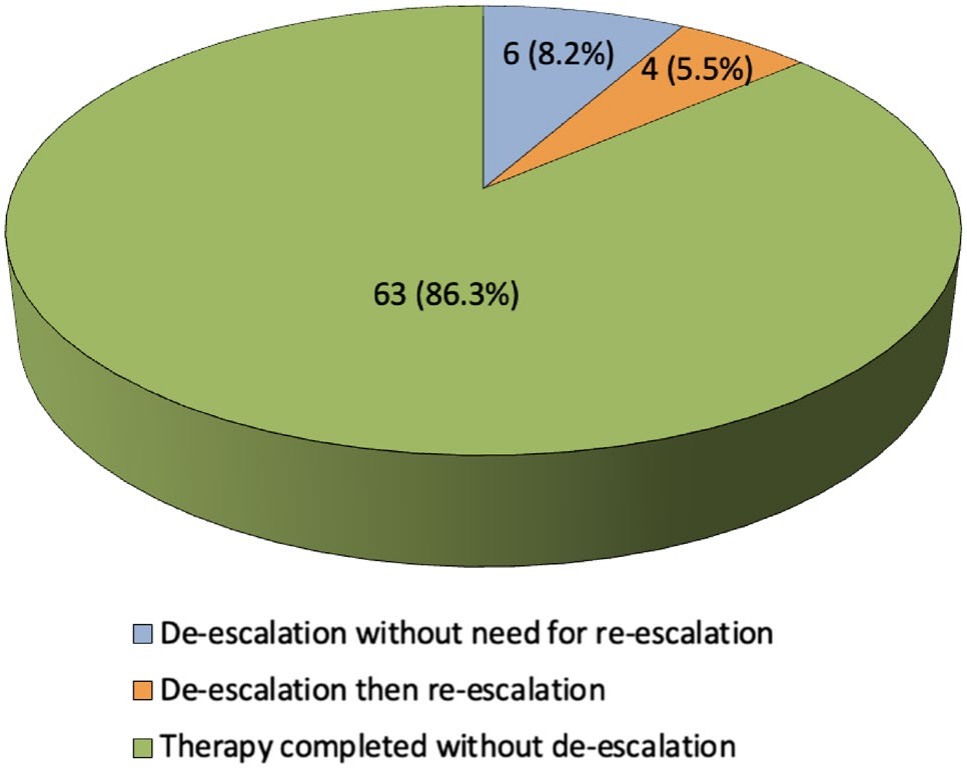
De-escalation/re-escalation status among ICU patients with COVID-19 (*N* = 73)

One hundred and nineteen critically ill patients with COVID-19 were screened, and 73 fitted the inclusion criteria. The mean ± SD age was 61.0 ± 19.4 years, with 31 (42.5%) male patients. Most patients, 60 (95.2%), were admitted to the medical ICU. The mean ± SD acute physiology and chronic health evaluation III (APACHE III) score were 19.0 ± 9.9, while the mean ± SD of sequential organ failure assessment (SOFA) score was 10.0 ± 6.7. Mechanical ventilation was used in 51 (69.9%) patients during ICU stay, for a mean ± SD of 20.1 ± 20.5 ventilation days (Table 1). Sixty-three (86.3%) patients completed their therapy without ADE. Among the ten patients in the ADE group (13.6%), four (5.5%) required re-escalation of treatment (Fig. 1). The clinical details of those patients can be found in the supplementary material (Table S1).

**Table 1:** Demographic and clinical characteristics of ICU patients with COVID-19 by ADE status

	De-escalation status		Total (*N* = 73)	*P* value
	No (*N* = 63)	Yes (*N* = 10)		
Age (years)^b^	59.1 ± 19.7	73.1 ± 12.1	61.0 ± 19.4	**0.033**
≤ 65^a^	36 (57.1%)	2 (20.0%)	38 (52.1%)	**0.041**
> 65^a^	27 (42.9%)	8 (80.0%)	35 (47.9%)	
Sex^a^				
Male	27 (42.9%)	4 (40.0%)	31 (42.5%)	> 0.99
Female	36 (57.1%)	6 (60.0%)	42 (57.5%)	
Weight^b^	158.8 ± 11.2	160.7 ± 12.3	159.1 ± 11.3	0.645
BMI^b^	32.2 ± 7.1	28.7 ± 4.9	31.8 ± 6.9	0.159
ICU admission type^a^				
Medical	60 (95.2%)	10 (100.0%)	70 (95.9%)	> 0.99
Surgical	3 (4.8%)	0 (0.0%)	3 (4.1%)	
Admission diagnosis^a^				
Respiratory failure	53 (84.1%)	9 (90.0%)	62 (84.9%)	> 0.99
Sepsis/septic shock	3 (4.8%)	3 (30.0%)	6 (8.2%)	**0.030**
Dialysis/AKI	4 (6.3%)	0 (0.0%)	4 (5.5%)	> 0.99
Another shock	3 (4.8%)	0 (0.0%)	3 (4.1%)	> 0.99
Post-surgery	1 (1.6%)	0 (0.0%)	1 (1.4%)	> 0.99
Others	4 (6.3%)	0 (0.0%)	4 (5.5%)	> 0.99
Comorbidity^a^				
Diabetes	37 (58.7%)	6 (60.0%)	43 (58.9%)	> 0.99
Hypertension	34 (54.0%)	5 (50.0%)	39 (53.4%)	> 0.99
Cardiovascular diseases (IHD, HF)	18 (28.6%)	3 (30.0%)	21 (28.8%)	> 0.99
Autoimmune diseases	12 (19.0%)	0 (0.0%)	12 (16.4%)	0.198
Solid organ transplantation	9 (14.3%)	1 (10.0%)	10 (13.7%)	> 0.99
Chronic kidney disease	8 (12.7%)	1 (10.0%)	9 (12.3%)	> 0.99
Non-hematologic malignancy	8 (12.7%)	0 (0.0%)	8 (11.0%)	0.588
End-stage renal disease	4 (6.3%)	3 (30.0%)	7 (9.6%)	**0.049**
Chronic lung disease (COPD/ ILD)	5 (7.9%)	0 (0.0%)	5 (6.8%)	> 0.99
Hematologic malignancy	3 (4.8%)	0 (0.0%)	3 (4.1%)	> 0.99
Liver disease	2 (3.2%)	0 (0.0%)	2 (2.7%)	> 0.99
Seizures disorder	1 (1.6%)	0 (0.0%)	1 (1.4%)	> 0.99
ICU scores^b^				
APACHE Score	17.9 ± 9.5	25.3 ± 10.3	19.0 ± 9.9	**0.048**
SOFA score	11.0 ± 7.1	9.3 ± 8.0	10.0 ± 6.7	> 0.99
Requiring RRT during ICU stay^a^	19 (30.2%)	5 (50.0%)	24 (32.9%)	0.281
Ventilation status at antibiotic initiation^a^				
Room air	10 (15.9%)	1 (10.0%)	11 (15.1%)	> 0.99
Low-flow nasal cannula	2 (3.2%)	2 (20.0%)	4 (5.5%)	0.088
High-flow nasal cannula	17 (27.0%)	1 (10.0%)	18 (24.7%)	0.434
Invasive ventilation	35 (55.6%)	6 (60.0%)	41 (56.2%)	> 0.99
Noninvasive ventilation	9 (14.3%)	0 (0.0%)	9 (12.3%)	0.345
Mechanical ventilation during ICU stay^a^	44 (69.8%)	7 (70.0%)	51 (69.9%)	> 0.99
Days of mechanical ventilation^b^	20.1 ± 20.7	19.6 ± 21.4	20.1 ± 20.5	0.773
COVID-19 status upon administration of broad-spectrum antibiotics^a^				
Positive	60 (95.2%)	10 (100.0%)	70 (95.9%)	> 0.99
Recovered	3 (4.8%)	0 (0.0%)	3 (4.1%)	
Duration of COVID infection (days)^b^	28.0 ± 18.1	29.1 ± 16.7	28.2 ± 17.7	0.668
*P*: *F* value upon broad-spectrum initiation^a^				
> 200	15 (25.4%)	3 (30.0%)	18 (26.1%)	0.128
100–200	25 (42.4%)	1 (10.0%)	26 (37.7%)	
< 100	19 (32.2%)	6 (60.0%)	25 (36.2%)	
Corticosteroids used^a^				
Any type	61 (96.8%)	9 (90.0%)	70 (95.9%)	0.362
Dexamethasone	51 (81.0%)	7 (70.0%)	58 (79.5%)	0.419
Methylprednisolone	16 (25.4%)	2 (20.0%)	18 (24.7%)	> 0.99
Hydrocortisone	22 (34.9%)	5 (50.0%)	27 (37.0%)	0.483
Tocilziumab use^a^	44 (69.8%)	7 (70.0%)	51 (69.9%)	> 0.99
Tocilizumab dose^a^				
One dose of 4 mg/kg	4 (9.1%)	1 (14.3%)	5 (9.8%)	0.730
One dose of 8 mg/kg	12 (27.3%)	3 (42.9%)	15 (29.4%)	
Two doses of 4 mg/kg	8 (18.2%)	1 (14.3%)	9 (17.6%)	
Two doses of 8 mg/kg	20 (45.5%)	2 (28.6%)	22 (43.1%)	
Laboratory investigations upon initiation of broad-spectrum antibiotics^a^				
d dimer (mg/L)	5.04 ± 12.70	3.09 ± 2.87	4.79 ± 11.88	0.800
Ferritin (μg/L)	848.3 ± 1414.5	690.6 ± 1092.5	827.4 ± 1370.2	0.544
CRP (nmol/L)	143.1 ± 136.0	98.7 ± 66.5	137.5 ± 129.9	0.727
ESR (mm/h)	70.2 ± 40.8	59.5 ± 68.6	67.1 ± 43.8	> 0.99

Statistically significant values are in bold

*AKI* acute kidney infection, *COPD* chronic obstructive pulmonary disease, *ICU* intensive care unit, *IHD* ischemic heart disease, *ILD* interstitial lung disease, *HF* heart failure

^a^*n* (%)

^b^Mean ± SD

Primary and secondary outcomes are summarized in Table 2. There was no statistically significant difference in the primary outcome, ICU mortality, between patients who continued antimicrobial therapy and those in the ADE group. ICU mortality occurred in 32 (43.8%) patients (Table 2). We found similar findings in all secondary outcomes (Table 2). Superinfection was found in 4 (5.4%) patients. Microorganisms found among superinfection patients are listed in Supplementary Table S2. Hospital mortality was observed in 33 (45.2%) patients. The mean ± SD length of ICU stay was 24.8 ± 20.7 days, while the mean ± SD length of hospital stay was 29.7 ± 22.9 days. Six (14.6%) patients had an event of ICU readmission within 30 days of ICU discharge. Recurrence of infection three months after antibiotic therapy was detected in 8 (32.0%) patients.

**Table 2:** Primary and secondary outcomes among ICU patients with COVID-19 by de-escalation status

	De-escalation status		Total (*N* = 73)	*P* value
	No (*N* = 63)	Yes (*N* = 10)		
Primary outcome^a^				
ICU mortality	26 (41.3%)	6 (60.0%)	32 (43.8%)	0.317
Secondary outcomes^a^				
Superinfection^a^	4 (6.3%)	0 (0.0%)	4 (5.4%)	> 0.99
Recurrence 3 months after completion of antibiotic therapy^a^	7 (38.9%)	1 (14.3%)	8 (32.0%)	0.493
Hospital mortality^a^	26 (41.3%)	7 (70.0%)	33 (45.2%)	0.169
Readmission to ICU within 30 days of ICU discharge^a^	5 (13.5%)	1 (25.0%)	6 (14.6%)	0.483
Length of ICU stay^b^	24.1 ± 20.9	28.7 ± 20.1	24.8 ± 20.7	0.369
Length of hospital stay^b^	29.3 ± 23.5	32.1 ± 19.6	29.7 ± 22.9	0.515

^a^*n* (%)

^b^Mean ± SD

Patients who continued on antibiotic therapy had a statistically significant longer duration of antibiotic use, with a mean ± SD of 9.4 ± 4.4 days in comparison to patients in the de-escalation group mean ± SD of 5.7 ± 3.1; *P* = 0.004 (Table 3). Meropenem was the most used antibiotic among 66 (90.4%) patients. Concurrent antibiotic use was found in ten (13.7%) patients, mainly fluoroquinolones 5 (6.8%) and aminoglycosides 4 (5.5%). The culture was positive in 35 (47.9%) of our patients. The most commonly reported microorganism was *Pseudomonas aeruginosa* in 7 (9.6%) cultures. The most seen resistance patterns were extended-spectrum beta-lactamase- and carbapenem-resistant *Pseudomonas* among 5 (14.3%) and 2 (5.7%) cultures, respectively (Table 4).

**Table 3:** Antibiotic use among ICU patients with COVID-19 by de-escalation status

	De-escalation status		Total (*N* = 73)	*P* value
	No (*N* = 63)	Yes (*N* = 10)		
Antibiotics used before ICU admission^a^	46 (73.0%)	4 (40.0%)	50 (68.5%)	0.063
Piperacillin tazobactam	23 (50.0%)	1 (25.0%)	24 (48.0%)	0.575
Ceftriaxone	9 (19.6%)	1 (25.0%)	10 (20.0%)	
Cefepime	6 (13.0%)	2 (50.0%)	8 (16.0%)	
Meropenem	2 (4.3%)	0 (0.0%)	2 (4.0%)	
Ceftazidime	2 (4.3%)	0 (0.0%)	2 (4.0%)	
Fluoroquinolones alone	2 (4.3%)	0 (0.0%)	2 (4.0%)	
Aminoglycosides	1 (2.2%)	0 (0.0%)	1 (2.0%)	
Others	1 (2.2%)	0 (0.0%)	1 (2.0%)	
Duration of use of antibiotics before ICU admission (days)^b^	3.3 ± 2.6	2.3 ± 1.5	3.2 ± 2.5	0.401
Primary broad-spectrum antibiotics^a^				
Meropenem	56 (88.9%)	10 (100.0%)	66 (90.4%)	0.583
Imipenem-cilastatin	3 (4.8%)	0 (0.0%)	3 (4.1%)	> 0.99
Ceftazidime-avibactam	2 (3.2%)	0 (0.0%)	2 (2.7%)	> 0.99
Ceftolozane-tazobactam	2 (3.2%)	0 (0.0%)	2 (2.7%)	> 0.99
Duration of use of primary broad-spectrum antibiotics (days)^b^	9.4 ± 4.4	5.7 ± 3.3	8.9 ± 4.4	**0.004**
Concurrently using antibiotics^a^				
Any antibiotic	9 (14.3%)	1 (10.0%)	10 (13.7%)	> 0.99
Fluoroquinolones	4 (6.3%)	1 (10.0%)	5 (6.8%)	> 0.99
Aminoglycosides	4 (6.3%)	0 (0.0%)	4 (5.5%)	> 0.99
Colistin	1 (1.6%)	1 (10.0%)	2 (2.7%)	0.257
Tigecycline	2 (3.2%)	0 (0.0%)	2 (2.7%)	> 0.99
Aztreonam	1 (1.6%)	0 (0.0%)	1 (1.4%)	> 0.99
Antibiotics resumed after discontinuation^a^	20 (32.8%)	0 (0.0%)	20 (32.8%)	NA
Duration before antibiotics was resumed after discontinuation (days)^b^	7.1 ± 5.2		7.1 ± 5.2	NA
Beta-lactam therapy streamlining to^a^				
Ceftriaxone		2 (20.0%)	2 (20.0%)	NA
Cefepime		3 (30.0%)	3 (30.0%)	
Fluoroquinolones alone		1 (10.0%)	1 (10.0%)	
Others		4 (40.0%)	4 (40.0%)	
Duration of therapy for streamlined antibiotic (days)^b^		6.8 ± 3.9	6.8 ± 3.9	NA
Duration of therapy after re-escalation (days)^b^		4.3 ± 3.3	4.3 ± 3.3	NA

^a^*n* (%)

^b^Mean ± SD

**Table 4:** Microbiologic findings among ICU patients with COVID-19 by de-escalation status

	De-escalation status		Total (*N* = 73)	*P* value
	No (*N* = 63)	Yes (*N* = 10)		
Cultures^a^				
Positive	27 (42.9%)	8 (80.0%)	35 (47.9%)	**0.036**
Negative	36 (57.1%)	2 (20.0%)	38 (52.1%)	
Culture site^a^				
Tracheal aspirate	14 (22.2%)	6 (60.0%)	20 (27.4%)	**0.021**
Urine	10 (15.9%)	1 (10.0%)	11 (15.1%)	> 0.99
Wound	5 (7.9%)	1 (10.0%)	6 (8.2%)	> 0.99
Blood	5 (7.9%)	0 (0.0%)	5 (6.8%)	> 0.99
Surgical drain	1 (1.6%)	1 (10.0%)	2 (2.7%)	0.257
Organism identified^a^				
Gram-positive organisms	9 (14.3%)	1 (10.0%)	10 (13.7%)	> 0.99
Gram-negative organisms	16 (25.4%)	5 (50.0%)	21 (28.8%)	0.139
Candida	6 (9.5%)	2 (20.0%)	8 (11.0%)	0.300
Organism name^a^				
*Pseudomonas aeruginosa*	6 (9.5%)	1 (10.0%)	7 (9.6%)	> 0.99
*Klebsiella pneumoniae*	6 (9.5%)	0 (0.0%)	6 (8.2%)	0.587
*Stenotrophomonas maltophilia*	3 (4.8%)	3 (30.0%)	6 (8.2%)	**0.030**
*Escherichia coli*	3 (4.8%)	1 (10.0%)	4 (5.5%)	0.453
*Candida albicans*	3 (4.8%)	1 (10.0%)	4 (5.5%)	0.453
*Staphylococcus aureus*	3 (4.8%)	0 (0.0%)	3 (4.1%)	> 0.99
*Staphylococcus epidermidis*	2 (3.2%)	1 (10.0%)	3 (4.1%)	0.362
*Acinetobacter baumannii*	1 (1.6%)	0 (0.0%)	1 (1.4%)	> 0.99
Coagulase-negative staphylococci	1 (1.6%)	0 (0.0%)	1 (1.4%)	> 0.99
Enterococci	1 (1.6%)	0 (0.0%)	1 (1.4%)	> 0.99
Non-albicans *Candida*	1 (1.6%)	0 (0.0%)	1 (1.4%)	> 0.99
*Aspergillus*	0 (0.0%)	1 (10.0%)	1 (1.4%)	0.137
Others	4 (6.3%)	0 (0.0%)	4 (5.5%)	> 0.99
Resistance^a^				
Extended-spectrum beta-lactamases	5 (18.5%)	0 (0.0%)	5 (14.3%)	0.497
Carbapenem-resistant *Pseudomonas*	2 (7.4%)	0 (0.0%)	2 (5.7%)	> 0.99
Carbapenem-resistant Enterobacteriaceae	0 (0.0%)	0 (0.0%)	0 (0.0%)	NA
None of the above	19 (70.4%)	8 (100%)	27 (77.1%)	0.189
History of multidrug-resistant or extensively-resistant organisms 6 months before ICU admission^a^				
Yes	3 (4.8%)	0 (0.0%)	3 (4.1%)	> 0.99

^a^*n* (%)

## Discussion

In our cohort of critically ill COVID-19 patients, only 10 (13.6%) patients underwent de-escalation, which is considered low compared with previously published studies [19, 22]. Microbiological confirmation was documented in 80 and 42.9% of patients in the ADE and continuation groups. Among the sixty-three patients whose antibiotics were continued without ADE, 27 (42.9%) had positive cultures. Sixteen of 27 had a susceptible gram-negative organism to the primary broad-spectrum antibiotic. Seven of these 16 cases (43.7%) had documented resistance requiring therapy continuation. A possible explanation for our cohort's low ADE percentage might be related to our hospital antibiogram. The high rate of multidrug resistance and extended-spectrum antibiotics might render prescribers to the de-escalation decision. Another reason might be the limited data on COVID-19 bacterial co-infection and the infection complication at the beginning of the pandemic [3, 23]. Optimization of diagnostic criteria might provide more objective guidance for ADE. [7] A recent Italian study that tested multiplex PCR assay on critically ill COVID-19 patients to aid in diagnosing pneumonia found that around 39 of 44 (88.6%) patients with positive samples had their antibiotic escalated or de-escalated [24].

In our study, superinfection was found only among patients who continued the therapy and accounted for 6.3% within this group. The overall superinfection rate in our study was relatively lower than in the previous study [22]. Previous studies of non-covid-19 patients detected more Superinfections in the streamlining group (27% in ADE versus 11% in non-ADE, *P* = 0.03) [10, 22, 25]. Superinfection in our study included two *Stenotrophomonas maltophilia* and two *Aspergillus* species. On the other hand, a recent retrospective study found that 43/989 (4.3%) COVID-19 patients had superinfection with *Pseudomonas aeruginosa* and *Escherichia coli* as the most isolated organisms [6].

There were no statistically significant differences in either ICU mortality (60 vs. 41.3%, *P* = 0.317) or hospital mortality (70 vs. 41.3%, *P* = 0.169) between ADE and continuation of the therapy groups, respectively. Indeed, higher APACHE III scores were found in the de-escalation group, 25.3 ± 10.3, compared to 17.9 ± 9.5 in the continuation group (*P* = 0.049). In many previously published studies, mortality was not different among non-COVID-19 patients whose antibiotics de-escalated or continued. However, a minority of the cases were severe or required ICU stay [22, 26]. In a systematic review and meta-analysis of 14 stewardship objectives, the relative risk reduction for mortality in the de-escalation group was 66% (RR 0·44, 95% CI 0.30–0.66, *P* < 0.0001) [27].

A randomized controlled study conducted on 120 septic patients found a trend for longer ICU length of stay with 9 [interquartile range (IQR) 5–22] days in the de-escalation group compared to 8 [IQR 4–15] days in the continuation group (*P* = 0.71). The mortality rate was similar between groups [22]. A position statement from a task force of the European Society of Intensive Care Medicine (ESICM) gave a moderate recommendation with low-quality evidence to consider both risks of death and difficulty in treating pathogens in the decision to de-escalating antibiotic therapy [28]. The main limitation of this study is the small sample size; we only had ten patients in the ADE group. Another limitation is restricting the definition of ADE to include a narrow spectrum of antibiotics only rather than having the early discontinuation or discontinuation of the combination therapy, including aminoglycosides or fluoroquinolones. Moreover, this cohort had no data on inflammatory marker levels or other radiological diagnostics.

## Conclusions

During the era of COVID-19, broad-spectrum antibiotics were only de-escalated in a small percentage of high disease severity score patients and microbiological confirmation. Broad-spectrum ADE in critically ill COVID-19 patients was not associated with higher mortality. The continual use of broad-spectrum antibiotics on COVID-19 patients leads to more superinfection cases in our hospital. A larger cohort is needed to confirm these findings.

## Supplementary Information

Below is the link to the electronic supplementary material.

Supplementary file1 (DOCX 15 kb)

## Data Availability

Any requested data will be available in a timely manner.

## Abbreviations

ADE: Antibiotic de-escalation
SARS-CoV-2: Severe acute respiratory syndrome coronavirus
IDSA: Infectious Diseases Society of America
SSC: Surviving sepsis campaign
NIH: National Institute of Health
KFSHRC-J: King Faisal Specialist Hospital and Research Center-Jeddah
APACHE III: Acute physiology and chronic health evaluation III
SOFA: Sequential organ failure assessment
ICU: Intensive care unit
COVID-19: Coronavirus disease-19
