# Supplementary material for: Continuation Versus De-escalation of Broad-Spectrum Antibiotic Therapy in Critically Ill COVID-19 Patients
Source: Dr. Sulaiman Al Habib Med J. 2023 Feb 28;5(2):33–41. doi: 10.1007/s44229-023-00027-0 (PMC9972303; doi:10.1007/s44229-023-00027-0)
Supplement: Supplementary file 1 — Supplementary file1 (DOCX 15 kb) [file 44229_2023_27_MOESM1_ESM.docx]

**Supplementary Table S1:** Characteristics and outcome for COVID-19 Patients whose broad-spectrum antibiotics re-escalated after de-escalation.

|  | **Patient 1** | **Patient 2** | **Patient 3** | **Patient 4** |
| --- | --- | --- | --- | --- |
| Age (years) | 49 | 88 | 76 | 82 |
| APACH III score | 35 | 19 | 35 | 35 |
| Cultures | Positive | Positive | Positive | Negative |
| Organism | Stenotrophomonas maltophilia | *Escherichia coli* | Stenotrophomonas maltophilia | NA |
| Broad-spectrum Antibiotic used | Meropenem | Meropenem | Meropenem | Meropenem |
| Duration (days) of therapy for broad-spectrum antibiotic | 3 | 6 | 6 | 5 |
| Beta-lactam therapy streamlining to | Trimethoprim/ sulfamethoxazole | Fluoroquinolones | Trimethoprim/ sulfamethoxazole | Ceftriaxone |
| Tocilizumab use | One dose of 8 mg/kg | Two doses of 4 mg/kg | One dose of 8 mg/kg | One dose of 4 mg/kg |
| ICU Mortality | Yes | Yes | Yes | Yes |
| Length of ICU stay (days) | 33 | 15 | 44 | 8 |
| Length of hospital stay (days) | 33 | 19 | 45 | 9 |

**Supplementary Table S2:** Organisms Identified in patients with superinfection

| **Patient** | **Organism Name** | **Site** |
| --- | --- | --- |
| 1 | *Stenotrophomonas maltophilia* | Tracheal aspirate |
| 2 | *Stenotrophomonas maltophilia* | Tracheal aspirate |
| 3 | Aspergillus species | Tracheal aspirate |
| 4 | *Candida albicans* / *Aspergillus* species | Tracheal aspirate |
